# Supplementary material for: Spontaneous neural synchrony links intrinsic spinal sensory and motor networks during unconsciousness
Source: eLife. 2021 May 27;10:e66308. doi: 10.7554/eLife.66308 (PMC8177891; doi:10.7554/eLife.66308)
Supplement: Transparent reporting form [file elife-66308-transrepform.pdf]

eLIFE
Westbrook Centre, Milton Road
Cambridge CB4 1YG
UK
P 01223 855340
W elifesciences.org
T @elife

***eLife's* transparent reporting form**

We encourage authors to provide detailed information *within their submission* to facilitate the interpretation and replication of experiments. Authors can upload supporting documentation to indicate the use of appropriate reporting guidelines for health-related research (see EQUATOR Network), life science research (see the BioSharing Information Resource), or the ARRIVE guidelines for reporting work involving animal research. Where applicable, authors should refer to any relevant reporting standards documents in this form.

If you have any questions, please consult our Journal Policies and/or contact us: editorial@elifesciences.org.

Sample-size estimation

- You should state whether an appropriate sample size was computed when the study was being designed
- You should state the statistical method of sample size computation and any required assumptions
- If no explicit power analysis was used, you should describe how you decided what sample (replicate) size (number) to use

Please outline where this information can be found within the submission (e.g., sections or figure legends), or explain why this information doesn't apply to your submission:

Detailed information about these aspects of the study can be seen in Materials and Methods, under the subheads "Discrimination of units, correlation and functional connectivity analyses," "Synthetic Data" and "Statistical methods."

Briefly, however, sample size determination is a complex process for data and analyses such as ours. Specifically, the number of animals per cohort, as is frequently taken to be the requisite sample size, is not the most appropriate measure of sample size for our study design. The ability to make statistical inferences about our primary outcome measure (i.e., correlation between neural spike trains) is based on the number of well-isolated neurons we were able to access per animal, per trial, per electrode. It is made more complicated because there exists no gold-standard 'benchmark' dataset for the specific questions asked.

Thus, we took a different approach to sample size determination than a standard a priori power analysis. Specifically, we used a two-fold validation method to enable statistical inference and ensure reproducibility and rigor. One phase of this approach involved replicating the experiments in an additional cohort of animals, while the other phase involved using the empirically acquired data as the basis for generating a large, statistically matched synthetic dataset. This dataset simulated the results of over 1,000 experiments per cohort, enabling generation of confidence intervals that could be used to compare to the empirically obtained data.

Details of the two animal cohorts are available in the following sections of the manuscript: "Materials and Methods," "Results -- *Functional connectivity within and between deep regions of the spinal gray matter is not abolished by preferential pharmacological depression*," and "Discussion." Details of the synthetic data are available in the following sections: "Materials and Methods" and "Discussion."

Replicates

- You should report how often each experiment was performed
- You should include a definition of biological versus technical replication

- The data obtained should be provided and sufficient information should be provided to indicate the number of independent biological and/or technical replicates
- If you encountered any outliers, you should describe how these were handled
- Criteria for exclusion/inclusion of data should be clearly stated
- High-throughput sequence data should be uploaded before submission, with a private link for reviewers provided (these are available from both GEO and ArrayExpress)

Please outline where this information can be found within the submission (e.g., sections or figure legends), or explain why this information doesn't apply to your submission:

Details of these aspects of the study are available in the Materials and Methods section, under the subheads "Discrimination of units, correlation, and functional connectivity analyses," "Synthetic Data" and "Statistical methods."

Statistical reporting

- Statistical analysis methods should be described and justified
- Raw data should be presented in figures whenever informative to do so (typically when N per group is less than 10)
- For each experiment, you should identify the statistical tests used, exact values of N, definitions of center, methods of multiple test correction, and dispersion and precision measures (e.g., mean, median, SD, SEM, confidence intervals; and, for the major substantive results, a measure of effect size (e.g., Pearson's r, Cohen's d)
- Report exact p-values wherever possible alongside the summary statistics and 95% confidence intervals. These should be reported for all key questions and not only when the p-value is less than 0.05.

Please outline where this information can be found within the submission (e.g., sections or figure legends), or explain why this information doesn't apply to your submission:

This information can be found in Materials and Methods, within each section of Results, within each figure/figure legend (including figure supplements for Figs. 5 and 7), and as Supplementary Materials.

(For large datasets, or papers with a very large number of statistical tests, you may upload a single table file with tests, Ns, etc., with reference to sections in the manuscript.)

Group allocation

- Indicate how samples were allocated into experimental groups (in the case of clinical studies, please specify allocation to treatment method); if randomization was used, please also state if restricted randomization was applied
- Indicate if masking was used during group allocation, data collection and/or data analysis

Please outline where this information can be found within the submission (e.g., sections or figure legends), or explain why this information doesn't apply to your submission:

This information can be found in Materials and Methods, within each section of Results, within each figure/figure legend.

Additional data files ("source data")

- We encourage you to upload relevant additional data files, such as numerical data that are represented as a graph in a figure, or as a summary table
- Where provided, these should be in the most useful format, and they can be uploaded as "Source data" files linked to a main figure or table
- Include model definition files including the full list of parameters used
- Include code used for data analysis (e.g., R, MatLab)
- Avoid stating that data files are "available upon request"

Please indicate the figures or tables for which source data files have been provided:

Source data is provided for Figures 2, 5, 6, 7, and 8, as are detailed statistical results tables.
